# Spatiotemporal regulation of human IFN-**ε** and innate immunity in the female reproductive tract

**DOI:** 10.1172/jci.insight.135407

**Published:** 2022-09-22

**Authors:** Nollaig M. Bourke, Sharon L. Achilles, Stephanie U-Shane Huang, Helen E. Cumming, San S. Lim, Irene Papageorgiou, Linden J. Gearing, Ross Chapman, Suruchi Thakore, Niamh E. Mangan, Sam Mesiano, Paul J. Hertzog

**Affiliations:** 1 Centre for Innate Immunity and Infectious Diseases, Hudson Institute of Medical Research, Clayton, Victoria, Australia.; 2 Discipline of Medical Gerontology, School of Medicine, Trinity Translational Medicine Institute, Trinity College Dublin, Dublin, Ireland.; 3 Department of Obstetrics, Gynecology, and Reproductive Sciences, University of Pittsburgh School of Medicine, Pittsburgh, Pennsylvania, USA.; 4 Magee-Womens Research Institute, Pittsburgh, Pennsylvania, USA.; 5 Department of Molecular and Translational Science, Monash University, Clayton, Victoria, Australia.; 6 Department of Obstetrics and Gynecology, University Hospitals of Cleveland, Cleveland, Ohio, USA.; 7 Department of Reproductive Biology, Case Western Reserve University School of Medicine, Cleveland, Ohio, USA.

**Keywords:** Immunology, Reproductive Biology, Cytokines

## Abstract

Although published studies have demonstrated that IFN-ε has a crucial role in regulating protective immunity in the mouse female reproductive tract, expression and regulation of IFN-ε in the human female reproductive tract (hFRT) have not been characterized to our knowledge. We obtained hFRT samples from a well-characterized cohort of women to enable us to comprehensively assess ex vivo IFN-ε expression in the hFRT at various stages of the menstrual cycle. We found that among the various types of IFNs, IFN-ε was uniquely, selectively, and constitutively expressed in the hFRT epithelium. It had distinct expression patterns in the surface and glandular epithelia of the upper hFRT compared with basal layers of the stratified squamous epithelia of the lower hFRT. There was cyclical variation of IFN-ε expression in the endometrial epithelium of the upper hFRT and not in the distal FRT, consistent with selective endometrial expression of the progesterone receptor and regulation of the *IFNE* promoter by progesterone. Because we showed IFN-ε stimulated important protective IFN-regulated genes in FRT epithelium, this characterization is a key element in understanding the mechanisms of hormonal control of mucosal immunity.

## Introduction

The human female reproductive tract (hFRT) mucosa is a unique site of immune regulation requiring robust responses against pathogenic infections yet maintaining tolerance with commensal bacteria, semen, and developing pregnancies (1). Epithelial cells lining the vagina, cervix, and uterus form an initial barrier against invading pathogens and are important regulators of immunity that have specialized capabilities, including antigen presentation and secretion of mucins, antimicrobial peptides, and chemokines that modulate recruitment and activation of the innate and adaptive immune cells (2). The fluctuation of sex hormones in women, primarily estradiol (E2) and progesterone, across the menstrual cycle may affect immune function in the hFRT, although the mechanisms mediating these effects are not comprehensively defined (3). During the progesterone-dominated luteal phase of menstrual cycle, when the endometrium prepares for fertilization and implantation, immune responses are relatively suppressed and a tolerogenic state is established (3). However, this environment may also be more permissible to pathogen invasion. For example, depot medroxyprogesterone acetate is commonly used in animal models, including HIV animal models, to induce higher susceptibility to *Chlamydia trachomatis* and herpes simplex virus 2 infections (4, 5). Factors that may mediate hormonal effects on host defense in the hFRT are not known. Antiviral and immunoregulatory cytokines, such as type I IFNs, are prime candidates (6).

The type I IFNs are a family of cytokines that include the conventional α and β subtypes, as well as the more recently identified IFN-ε. All type I IFNs bind IFN-α receptors 1 and 2, activate JAK/STAT signaling, and regulate the expression of potentially thousands of IFN-regulated genes (IRGs) (7). The effector proteins encoded by IRGs can modulate a wide range of biological responses, including antiviral activities, cell cycle regulation, survival and apoptosis, immune effector cell activity, and chemotaxis. Epithelial cells that line the vaginal, cervical, and endometrial mucosa are key sentinels that produce conventional type I IFNs (e.g., α and β subtypes) upon pathogen challenge (2). IFN-ε, which we characterized in the mouse female reproductive tract (mFRT) to be constitutively expressed in the endometrium and protective against viral and bacterial sexually transmitted infections (STIs) (8), may have an important role in STI protection in the mFRT. We determined that unlike conventional type I IFNs, murine IFN-ε was not induced by pattern recognition receptor pathways. Interestingly, we found that IFN-ε was hormonally regulated and IFN-ε levels fluctuated in the endometrium across the estrus cycle. Furthermore, in vitro experiments conducted with human cells demonstrated that IFN-ε can block HIV replication at several steps of viral replication by induction of antiviral IRGs (9, 10). The expression, distribution, regulation, and functions of IFN-ε in the hFRT are currently unknown. Therefore, we designed and conducted a cross-sectional study of healthy reproductive-aged women with normal menstrual cycles to characterize IFN-ε expression in the hFRT during the follicular and luteal phases of menses.

## Results

### Participant enrollment and demographic characteristics.

The 33 enrolled, eligible participants in cohort 1 were allocated to follicular-phase or luteal-phase analysis groups on the basis of serum progesterone concentration measured by ultra-high-performance liquid chromatography tandem mass spectrometry (UPLC/MS/MS). We defined follicular and luteal phases of menses as serum progesterone concentration < 1,000 pg/mL and > 2,000 pg/mL, respectively. The demographic characteristics did not differ by allocated menstrual-phase arm (Table 1). The median serum progesterone concentrations were 49 (IQR 39.5, 318.5) and 6171 (IQR 2998, 9774) pg/mL for participants in the follicular and luteal analysis groups, respectively.

### IFN-ε is expressed throughout the hFRT.

**Figure 1:**
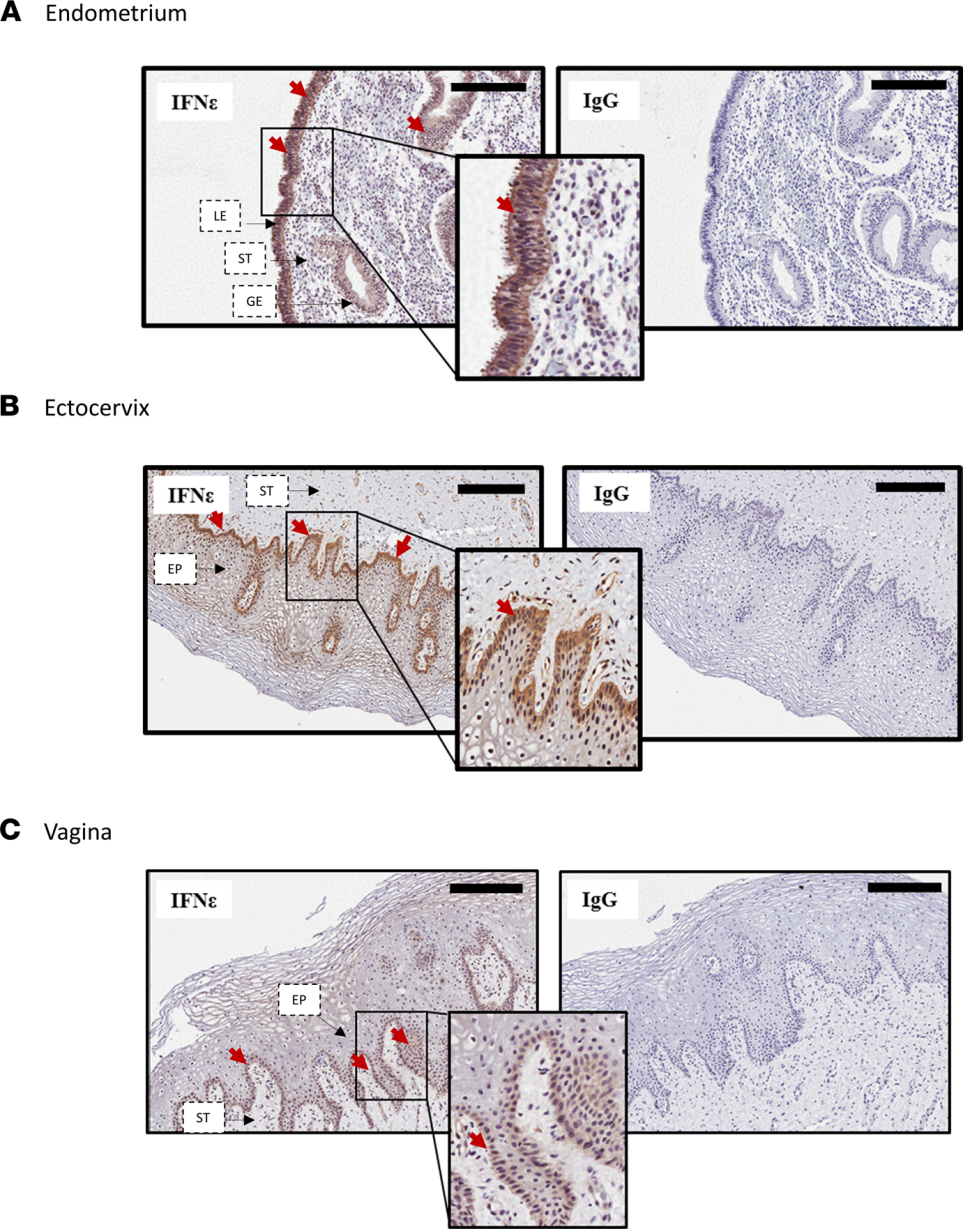
Distinct expression patterns of IFN-ε in upper and lower hFRT mucosa. (**A**–**C**) Representative images of IFN-ε expression in sections from matched biopsy samples from 33 women. Sections from the (**A**) endometrium, (**B**) ectocervix, and (**C**) vagina were stained for expression of IFN-ε (brown staining, highlighted with red arrows) or IgG control. Scale bar, 200 μm. EP, epithelium, GE, glandular epithelium; LE, luminal epithelium; ST, stroma.

IHC evaluation of matched vaginal, ectocervical, and endometrial biopsy samples from all participants in cohort 1 demonstrated that IFN-ε was highly expressed in the stratified squamous epithelium localized to the basal and parabasal layers of the lower hFRT (i.e., vagina and ectocervix) (Figure 1, B and C). In the endometrium, strong IFN-ε staining was detected in both the luminal and glandular epithelium (Figure 1A). Thus, IFN-ε is constitutively expressed appropriately to exert physiological functions locally throughout the hFRT.

### Selective cyclic variation of endometrial IFN-ε.

**Figure 2:**
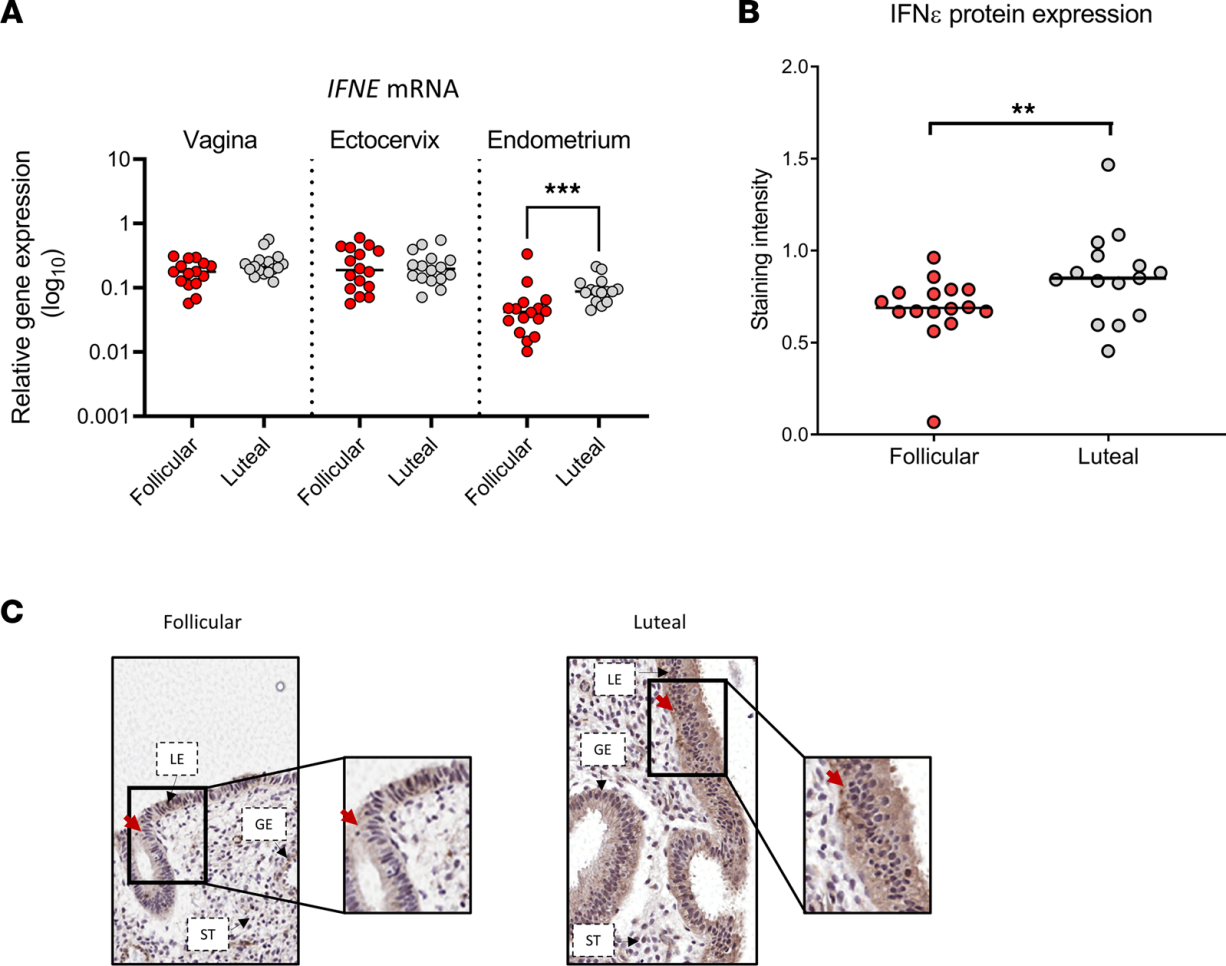
Cyclic variation of IFN-ε expression only in the upper hFRT. (**A**) *IFNE* mRNA expression, as determined by quantitative PCR (qPCR), in vaginal, ectocervical, and endometrial biopsy samples stratified into follicular (*n* = 16) and luteal (*n* = 16) stages of the menstrual cycle. Quantification (**B**) and representative IHC images (**C**) of endometrial epithelial IFN-ε staining intensity in women in the follicular or luteal stage of the menstrual cycle, using the Aperio positive pixel–count algorithm to generate intensity values for staining. IFN-ε staining is highlighted with red arrows. Significance was determined using either Kruskal-Wallis testing with Dunn’s multiple-comparison analysis (**A**) or Mann-Whitney *U* test (**B**). **P < 0.01; ***P < 0.001. GE, glandular epithelium; LE, luminal epithelium; ST, stroma.

Examination of IFN-ε expression in the endometrium at the spatial and molecular levels in cohort 1 revealed that there was significantly higher *IFNE* mRNA abundance in the endometrium during the luteal phase of the menstrual cycle compared with expression during the follicular phase (*P* < 0.001) (Figure 2A). Similarly, there was a significantly higher level of IFN-ε protein in the epithelium of the endometrium during the luteal phase of the cycle compared with the follicular phase (*P* < 0.05) (Figure 2, B and C). There were no differences in vaginal and ectocervical *IFNE* mRNA (Figure 2A) or IFN-ε protein by menstrual cycle phase (Supplemental Figure 1, A and B; supplemental material available online with this article; https://doi.org/10.1172/jci.insight.135407DS1).

### IFN-ε expression is negatively regulated by progesterone receptor.

**Figure 3:**
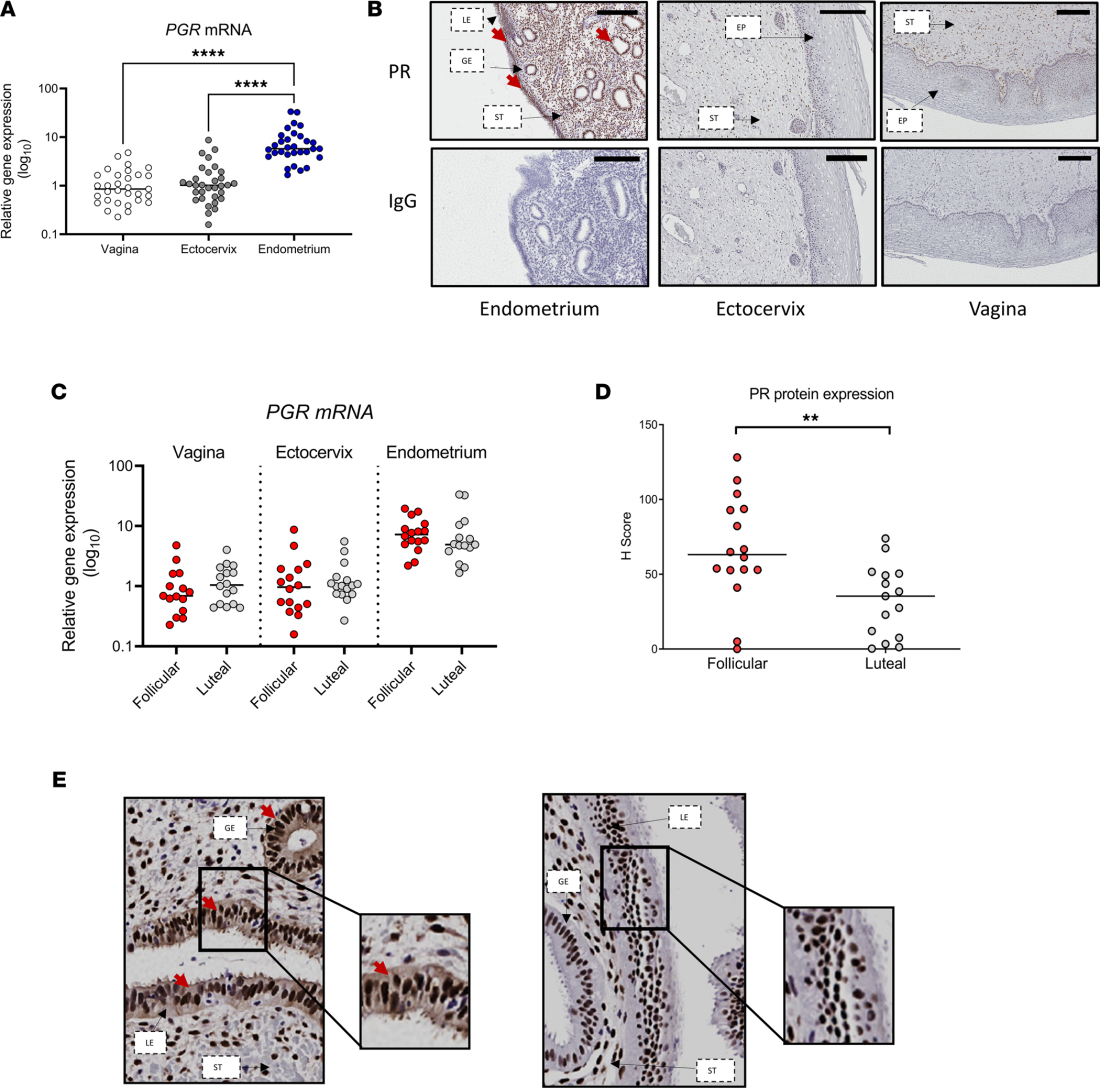
Expression of PR selective and cyclic changes only in upper hFRT. (**A**)PR gene (*PGR*) mRNA expression, as determined by qPCR, in vaginal, ectocervical, and endometrial biopsy samples. (**B**) Representative images of PR expression in sections from matched biopsy samples from 33 women. Sections from the endometrium, ectocervix, and vagina were stained for expression of PR (brown) or IgG control. Scale bar, 200 μm. (**C**) *PGR* mRNA expression, as determined by qPCR, in vaginal, ectocervical, and endometrial biopsy samples stratified into follicular (*n* = 16) and luteal (*n* = 16) stages of the menstrual cycle. (**D**) Quantification and (**E**) representative IHC images of cytoplasmic PR staining intensity in endometrial epithelial cells from women in the follicular or luteal stage of the menstrual cycle. H-scores for staining were generated using the Aperio cytoplasm algorithm, which classifies cytoplasmic staining intensity scoring as 0, none; 1+, weak; 2+ moderate; or 3+, strong, and uses this to generate an H-score using the following formula: 1 × (%1+) + 2 × (%2+) + 3 × (%3+). PR staining is highlighted with red arrows. Significance determined using either Kruskal-Wallis testing with Dunn’s multiple-comparison analysis (**A** and **C**) or Mann-Whitney *U* test (**D**). **P < 0.01, ****P < 0.0001. EP, epithelium, GE, glandular epithelium; LE, luminal epithelium; ST, stroma.

Because variation of IFN-ε expression in the menstrual cycle occurred only in the upper hFRT, we examined the expression patterns of hormone receptors that might mediate this differential regulation in this cohort. We found high expression of progesterone receptor (PR) transcript (Figure 3A) in the endometrium compared with very low expression of PR transcript in the ectocervix and vagina. Accordingly, IHC analysis of PR protein (Figure 3B) across the hFRT revealed strong staining of PR in both luminal and glandular endometrial epithelial cells, but it was barely detectable in the ectocervix and vagina. Although progesterone receptor gene (*PGR*) mRNA was not evidently different in the endometrium, on the basis of cycle stage (Figure 3C), the endometrial PR protein level was significantly lower during the luteal stage of the menstrual cycle (*P* < 0.01) (Figure 3, D and E). These changes were particularly evident in the epithelial cytoplasmic PR abundance

**Figure 4:**
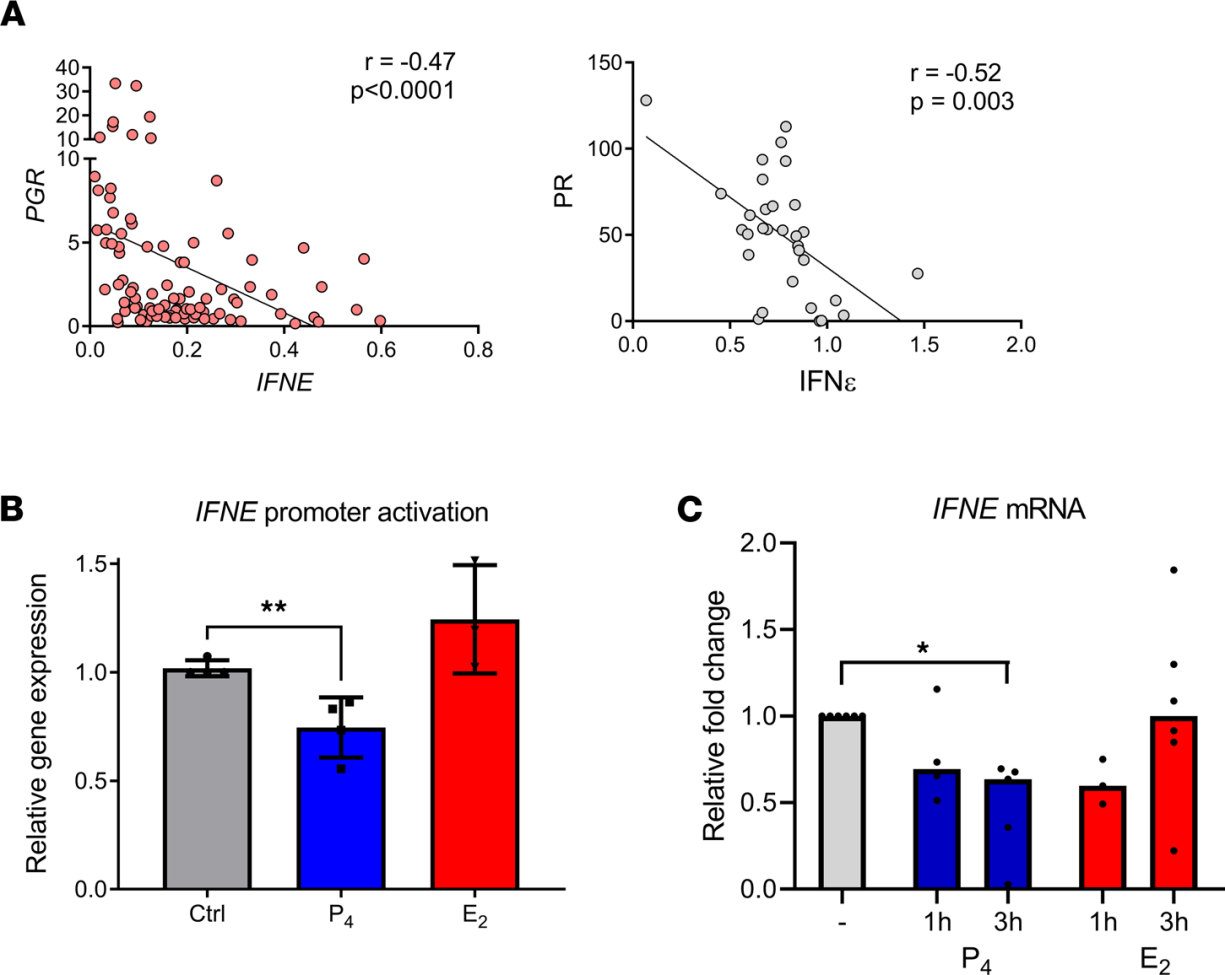
Regulation of IFN-ε by PR. (**A**) Negative correlation of both mRNA (left) and protein expression (right) of IFN-ε and PR in hFRT cells. Spearman correlation analysis. (**B**) Luciferase reporter assay measuring activation of the human *IFNE* promoter in ECC-1 cells after treatment with either 10 nM progesterone or 10 nM estrogen for 4 hours. Data are from 4 independent biological replicates, each in technical triplicate, shown as mean +SEM and analyzed using Student’s 2-tailed *t* test. **P < 0.01. (**C**) Primary uterine epithelial cells were isolated from endometrial biopsy specimens (from up to 6 donors) and cultured for 3 days prior to stimulation for either 1 or 3 hours with 10 nM progesterone or 10 nM estrogen. *IFNE* expression was quantified using qPCR, expressed relative to expression of *18S* and fold change relative to unstimulated control. Significance was determined using Kruskal-Wallis testing with Dunn’s multiple-comparison analysis. *P < 0.05.

Regression analysis of our independent measures of PR and IFN-ε expression levels for individual samples showed an inverse correlation that was statistically significant at both the mRNA and protein levels (*P* < 0.01) (Figure 4, A and B, respectively). We found no correlation between *IFNE* and *ESR1* expression (Supplemental Figure 2, A and B).

To investigate whether this inverse correlation was due to a direct suppression of IFN-ε expression by PR, we used an endometrial epithelial cell line, ECC-1, to characterize the regulation by progesterone of a luciferase gene construct under the control of the human *IFNE* promoter. In this in vitro system, progesterone stimulation significantly inhibited activation of the human *IFNE* promoter (*P* < 0.001) (Figure 4B) and estrogen stimulation had no effect on promoter activity. To confirm this finding in primary uterine epithelial cells, we cultured cells from endometrial biopsies obtained from cohort 2, stimulated them with progesterone or E2 and assessed *IFNE* expression. In this ex vivo model, *IFNE* expression significantly decreased after 3 hours of in vitro stimulation with progesterone (*P* < 0.05) (Figure 4C), whereas in vitro stimulation with estrogen did not alter *IFNE* expression, despite these cells being responsive to estrogen stimulation (Supplemental Figure 2C). These data demonstrated a negative regulation of human IFN-ε expression by progesterone.

### IFN-ε regulates protective immunoregulatory pathways in the hFRT.

**Figure 5:**
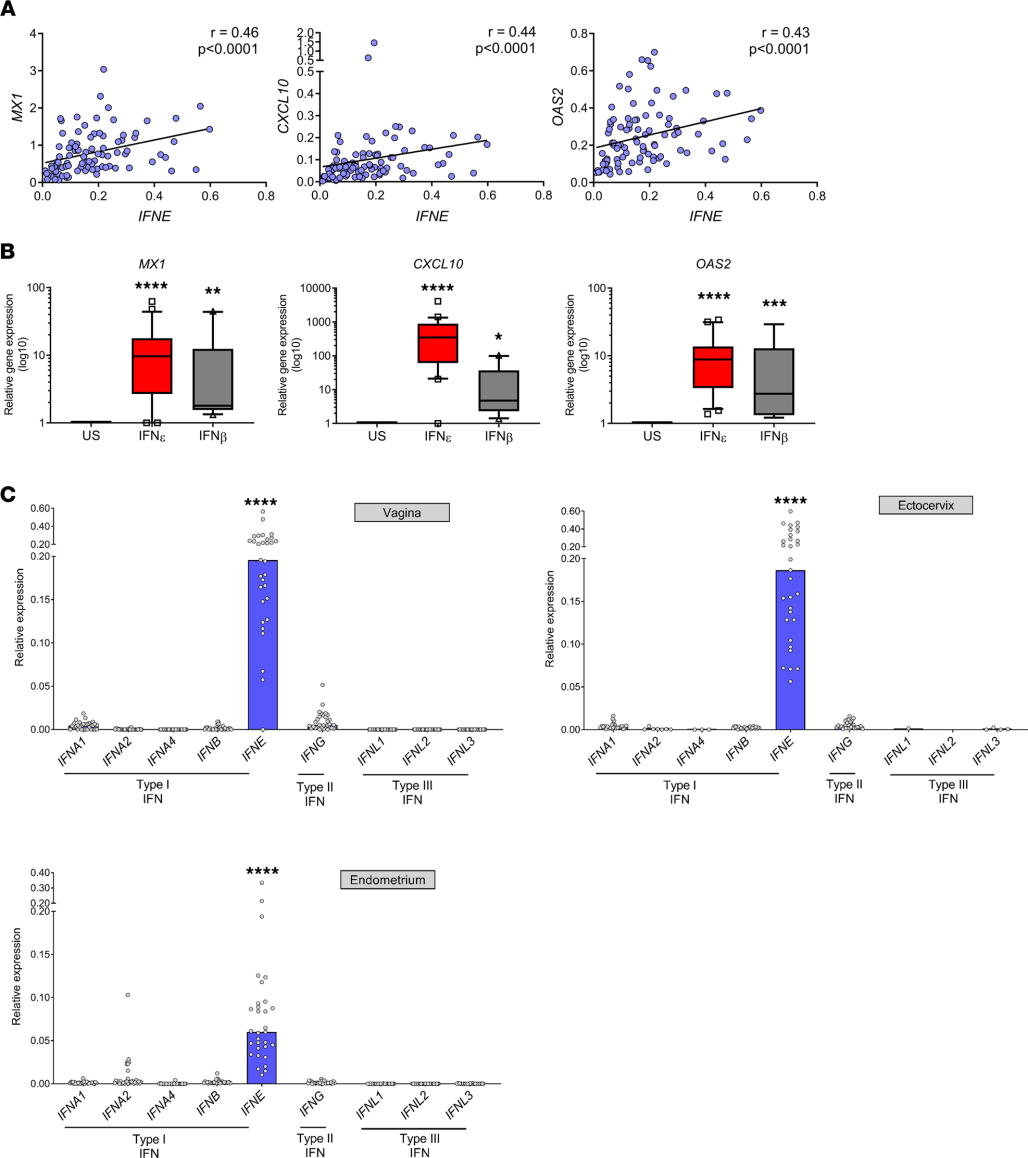
Exclusive expression of IFN-ε in hFRT regulates immune-protective IRGs. (**A**) Spearman’s correlation analysis of the expression of *IFNE* with the IRGs *MX1*, *CXCL10*, and *OAS2* across hFRT biopsy samples. (**B**) Primary uterine epithelial cells were isolated from endometrial biopsy specimens and cultured for 3 days prior to stimulation with IFN-β (*n* = 9) or IFN-ε (*n* = 20). (**C**) Expression of type I IFN (*IFNA1*, *IFNA2*, *IFNA4*, *IFNB*, *IFNE*), type II IFN (*IFNG*), and type III IFN (*IL28A*, *IL28B*, *IL29*) was quantified by qPCR in matched vaginal, ectocervical, and endometrial biopsy samples from 32 women regardless of phase of menses. In vaginal samples, *IFNA1* was not detectable (N/D) in 10, *IFNA2* was N/D in 24, *IFNA4* was N/D in 31, *IFNB* was N/D in 13, *IFNG* was N/D in 2, *IFNL1*and *IFNL2* were N/D in 31, and *IFNL3* was N/D in 30 specimens. In ectocervical samples, *IFNA1* was N/D in 6, *IFNA2* was N/D in 24, *IFNA4* was N/D in 28, *IFNB* was N/D in 7, *IFNG* was N/D in 1, *IFNL1* was N/D in 30, *IFNL2* was N/D in 31, and was *IFNL3* was N/D in 27 specimens. In endometrial samples, *IFNA1* was N/D in 10, *IFNA2* was N/D in 24, *IFNA4* was N/D in 31, *IFNB* was N/D in 13, *IFNG* was N/D in 2, *IFNL1* and *IFNL2* was N/D in 31, and *IFNL3* was N/D in 30 specimens. Gene expression was quantified using qPCR, normalized to *18S* expression, and expressed relative to untreated control cells. The box plots depict the minimum and maximum values (whiskers), the upper and lower quartiles, and the median. The length of the box represents the interquartile range. Data were analyzed using Kruskal-Wallis testing with Dunn’s multiple-comparison analysis: ****P < 0.0001; or Mann-Whitney *U* test: *P < 0.05, **P < 0.01, ***P < 0.001. US, unstimulated.

To determine whether this constitutive, hormone-regulated IFN-ε was active in modulating immune responses in the hFRT, we measured the expression of IRGs that encode known immune effector and signaling proteins in cohort 1 samples. All samples collected, regardless of phase of menses or sample type (vaginal, cervical, or endometrial), demonstrated strong and significant correlation of *IFNE* mRNA expression with expression of antiviral IRGs (namely, *MX1* *OAS2*, *IRF7*), chemokines (*CXCL10*), pathogen-sensing IRGs (*DDX58*), and IFN signaling (*STAT1*) (Figure 5A and Supplemental Figure 3A).

To confirm that IFN-ε directly induces innate immune effector molecules, we stimulated vaginal epithelial cells (a VK2 cell line), ectocervical epithelial cells (an Ect1 cell line) (Supplemental Figure 3B), and uterine epithelial cells (primary cells cultured from endometrial biopsy samples from cohort 2; Figure 5B) with exogenous IFN-ε. The IRGs *MX1*, *OAS2*, and *CXCL10* were substantially induced approximately 10-fold to 100-fold.

### IFN-ε is the sole IFN expressed constitutively in the hFRT.

Although these data are consistent with the hypothesis that basal expression of IFN-ε in the FRT constitutively maintains innate immune responses at this site, many IFNs could regulate these types of responses. Until now, however, the relative expression of all IFNs in the FRT has not been comprehensive and conclusively examined, to our knowledge. This study reveals that *IFNE* was selectively and strongly expressed in the vagina, ectocervix, and endometrium relative to expression of other type 1 (*IFNA1*, *IFNA2*, *IFNA4*, *IFNB*), type II (*IFNG*), and type III (*IFNL1*, *IFNL2*, *IFNL3*) IFNs (*P* < 0.0001), each of which was either undetectable or had very low expression compared with *IFNE* expression (Figure 5C).

Therefore, these data constitute a compelling case that IFN-ε is the predominant driver of IFN-dependent immunity in homeostatic conditions in the FRT, based on the observations that (a) IFN-ε expression levels correlate significantly with induction of important immunoregulatory genes; (b) these genes were independently demonstrated to be inducible by direct action of IFN-ε on FRT epithelial cells; and (c) IFN-ε is essentially the only IFN expressed across the FRT.

### IFN-ε protein expression is detectable in cervicovaginal lavage fluid.

**Figure 6:**
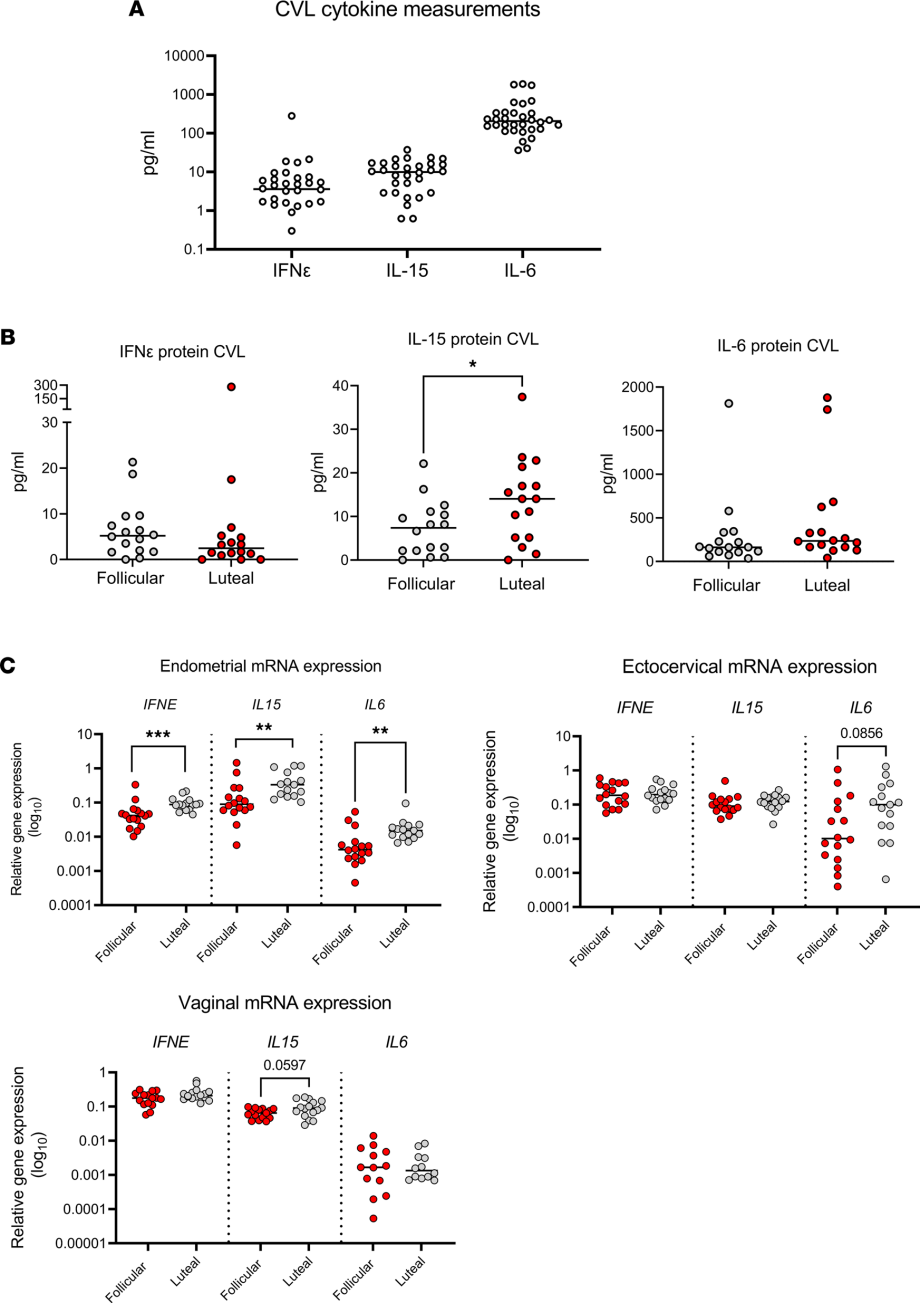
IFN-ε protein is expressed in CVL. (**A**) Concentrations of IFN-ε, IL-15, and IL-6 were quantified in CVL fluid (*n* = 32) using either laboratory-developed (IFN-ε) or commercially available (IL-15, IL-6) immunoassays. There was undetectable cytokine expression in 4 samples for IFN-ε and 2 samples for IL-15. (**B**) CVL IFN-ε, IL-15, and IL-6 expression was stratified by cycle stage into follicular stage samples (*n* = 16) and luteal stage samples (*n* = 16). There was undetectable cytokine expression for IFN-ε in 1 follicular and 3 luteal stage samples and IL-15 in 1 follicular and 1 luteal stage sample. (**C**) *IFNE*, *IL15*, and *IL6* mRNA expression, as determined by qPCR, in endometrial, ectocervical, and vaginal biopsy samples stratified into follicular (*n* = 16) and luteal (*n* = 16) stages of menstrual cycle. Mann-Whitney *U* tests were applied to determine cyclic differences for each gene or protein of interest. *P < 0.05, **P < 0.01,***P < 0.001.

We developed a sandwich ELISA using in-house monoclonal Abs to detect significant levels of IFN-ε production in matched cervicovaginal lavage (CVL) samples collected from cohort 1, confirming IFN-ε protein expression in FRT secretions (Figure 6A). We compared levels of IFN-ε with those of 2 other cytokines known to be expressed in CVL: IL-15 and IL-6. IFN-ε levels in CVL were similar to IL-15 levels (0–20 pg/mL); IL-6 expression was notably higher than both of these cytokines (Figure 6A). Interestingly, we did not find that IFN-ε expression in CVL samples differed on the basis of menstrual cycle stage, similar to IL-6 and unlike IL-15, levels of which were higher in CVL samples from women in the luteal stage of their cycle compared with the follicular stage (Figure 6B). This was despite evident hormonal regulation of *IFNE*, *IL15*, and *IL6* mRNA in endometrial biopsy samples, where expression of all 3 transcripts was significantly higher in the luteal stage of the cycle (Figure 6C). Similar to *IFNE*, neither *IL15* nor *IL6* mRNA expression in the ectocervix or vagina differed significantly on the basis of cycle stage. Endometrial *IL15* mRNA expression did correlate with IL-15 levels in CVL (Spearman *r* = 0.44; *P* = 0.01), yet endometrial *IFNE* or *IL6* mRNA expression did not correlate with levels of these cytokines in CVL. Furthermore, the epithelial origin of IFN-ε in the FRT is confirmed by its detection in lysates of cultured FRT-derived cancer cell lines (data not shown).

## Discussion

This study provides characterization of the spatiotemporal expression and hormonal regulation of IFN-ε in distinct parts of the hFRT from a well-characterized cohort of 33 women. Importantly, the study design enabled evaluation of IFN-ε expression throughout the hFRT and these data further our previous mechanistic studies conducted in mouse models. Here we demonstrate that IFN-ε expression (assessed by mRNA and protein abundance) is contiguously expressed from the lower to the upper hFRT at major sites requiring immune protection and is also detectable in hFRT secretions. IFN-ε is highly expressed in the luminal and glandular epithelium of the human endometrium, the site of implantation and of immune importance for protection against ascending infections such as *Chlamydia*. There was also stronger expression of IFN-ε in the basal layers of the stratified squamous epithelium of the cervix and vagina, which are important sites of infection with viruses such as HIV, whose replication we and others have demonstrated can be inhibited by IFN-ε (9, 10).

A clear finding in this study was that *IFNE* was the sole IFN (type I, II, or III) measured that was substantially, consistently, and constitutively expressed throughout the hFRT. IFN-ε expression in CVL samples was similar to the concentrations of IL-15 detected in this sample type. IFN-ε is, therefore, a prime candidate for regulating homeostatic signals to fine-tune the innate immune system in the hFRT mucosal surfaces. The efficacy of IFN-ε regulation of homeostatic immunity was shown by the demonstrationthat IFN-ε can induce IRGs with important functions. We further demonstrated that immunoregulatory IRG expression in hFRT samples was strongly correlated with the high *IFNE* expression in the hFRT. These data agree with our previously reported mouse model data study showing that IFN-ε^–/–^ mice have reduced IRG expression in the mFRT (8). “Basal” constitutive expression of regulatory IRGs that can modulate cellular processes such as metabolism, differentiation, proliferation, survival, and angiogenesis, in addition to their more prominent protective role in viral and bacterial infection and general immunoregulation, may be a crucial role for this unique IFN in tonic signaling to tune the mucosal innate immune system.

The data herein demonstrate that *PGR* is predominantly expressed in the endometrium and that *IFNE* expression is suppressed by progesterone, suggesting that IFN-ε production is hormonally regulated in the hFRT. Surprisingly, this regulation appears to occur in the endometrium and not in the vagina or ectocervix. This is likely because *PGR* expression in high in endometrial cells compared with cells in the vagina and ectocervix. Indeed, there was a substantial and significant inverse correlation between IFN-ε and PR expression at the mRNA and protein levels across all individuals in this study. PR expression is itself regulated by E2, and studies are warranted to decipher the complex relationships among estrogen responses, PR regulation, and IFN-ε expression in endometrial epithelial cells. Although we did not observe hormonal regulation in the lower hFRT, *IFNE* expression has been previously demonstrated to be upregulated in the human ectocervix upon exposure to semen (11, 12) and has been hypothesized to have an immunomodulatory mechanism within the hFRT to reduce risk of infection by HIV (13).

### Strengths and limitations.

A major strength of this work was that the data generated on ex vivo IFN-ε expression and associated hormonal responses (cohort 1), with the exception of the cell line and primary uterine epithelial cell work (cohort 2), were taken from a tightly controlled clinical study with strict inclusion and exclusion criteria to ensure we could account for many confounding factors, such as infections, medications, and exogenous hormonal influences, that many other studies based on FRT samples have not done. Each participant had matched samples taken from the FRT at time of enrolment, including matched vaginal, cervical, and endometrial biopsy samples, as well as cervical lavages. We performed mass spectrometry analysis to confirm cycle stage rather than rely solely on self-reported and/or histological approaches. For all IHC analyses, we created tissue arrays, with each slide containing matched vaginal, cervical, and endometrial tissue sections from up to 9 participants in our study, thus ensuring that all IHC staining was carefully controlled to minimize inter- and intraindividual variation. We revealed the relationship between IFN-ε abundance in the endometrium and progesterone responses, but more work is required to conclusively investigate the specific mechanisms of this relationship and what this may mean for women using hormone-based contraceptives.

### Conclusion.

IFN-ε likely has an important defensive role in the hFRT against pathogens, because it is constitutively expressed in the ectocervix and throughout the hFRT and regulates immune modulation in hFRT epithelium. Our results demonstrating IFN-ε suppression via the PR warrant additional study to evaluate if use of hormonal contraceptives with high affinity for the PR may also suppress IFN-ε and whether this may affect susceptibility to STIs, including HIV.

## Methods

### Participant recruitment and sampling

#### Cohort 1.

We performed a cross-sectional study (ClinicalTrials.gov identifier NCT02416154) at the Magee-Womens Research Institute (Pittsburgh, Pennsylvania, USA) of healthy, reproductive-aged women with normal menstrual cycles who were free of exogenous hormonal contraceptives. The University of Pittsburgh IRB and the Monash Health Human Research Ethics Committee both approved this study. All participants were enrolled at the Center for Family Planning Research at UPMC Magee-Womens Hospital and signed informed consent documents before study participation.

Being free of exogenous steroid hormones and in a defined (follicular versus luteal) phase of menses was central to the study design; therefore, laboratory confirmation by UPLC/MS/MS was performed to evaluate serum progesterone and E2, as well as a panel of synthetic progestins that cover the majority of regionally available contraceptive progestins.

Between August 2015 and August 2016, 44 participants were assessed for study eligibility, and 34 women, aged 18–35 years, were enrolled. Of the enrolled participants, 17 were in the follicular phase and 16 were in the luteal phase of the menstrual cycle; 1 participant was discontinued after enrollment for a positive screening test for *C. trachomatis*. Eligible women were healthy, were HIV negative, were not pregnant, and reported regular menstrual cycles every 21–35 days. Women were excluded if, within 30 days of enrollment, they (a) used any hormonal or intrauterine contraceptive methods; (b) underwent any surgical procedure involving the pelvis (including biopsy); (c) were diagnosed with any urogenital tract infection; and (d) used any vaginal or systemic antibiotics, oral or vaginal steroids, or any vaginal product or device (including spermicide, microbicide, douche, sex toy, cervical cap, menstrual collection device, diaphragm, or pessary) except tampons and condoms. Women were also excluded if they used depot medroxyprogesterone acetate within 10 months of enrollment, were pregnant or breastfeeding within 60 days of enrollment, or had a new sex partner within 90 days of enrollment. Exclusion criteria included having unprotected heterosexual intercourse since last reported menses, having vaginal or anal intercourse within 36 hours prior to the enrollment study visit, having a prior hysterectomy or malignancy of the cervix or uterus, and having any history of immunosuppression, including immunosuppression associated with chronic disease.

Medical, gynecologic, and sexual histories were obtained, and screening procedures were conducted, including urine pregnancy testing; rapid HIV screening (OraQuick, OraSure Technologies); collection of genital tract swabs for detection of *Neisseria gonorrhoeae* and *C*. *trachomatis* (Hologic Inc.); and rapid testing for *Trichomonas vaginalis* (OSOM, Sekisui Diagnostics). Participants were enrolled on the same day as screening when all eligibility criteria were met, including no vaginal bleeding on examination and being in the follicular (days 3–12) or luteal (~10 days prior to anticipated start of menses) phase of their menstrual cycle by self-report. Given the low-risk study population, participants with no clinical signs of genital infection were enrolled with pending screening tests and were discontinued after enrollment if a screening test rendered them ineligible. Final group allocation to phase of menses was based on UPLC/MS/MS serum hormone analysis, with follicular phase serum progesterone concentration < 1,000 pg/mL and luteal phase serum progesterone concentration > 2,000 pg/mL.

Five biopsy samples from the genital tract were obtained per participant: 2 vaginal biopsy samples from the upper vagina, 2 cervical biopsy samples from the squamocolumnar junction, and 1 endometrial biopsy sample. Vaginal and cervical biopsy samples were obtained with a standard gynecologic biopsy instrument and each measured approximately 2 × 3 × 2 mm. The endometrial biopsy specimen was obtained using a standard endometrial sampler (Pipelle, Cooper Surgical). Participants were given the option of a cervical anesthetic injection with 10 mL of 1% lidocaine solution prior to the endometrial biopsy. If this was elected, it was administered after the vaginal and cervical biopsy samples were obtained. One of each of the vaginal and cervical biopsy samples and half of the endometrial biopsy sample were each placed in 1 mL of RNA*later* (Thermo Fisher Scientific) and stored at 4°C overnight before being transferred to storage at −80°C. The second vaginal and cervical biopsy samples and the remaining half of the endometrial biopsy sample were separately placed into histology cassettes and incubated in 10% formalin fixative for 24 hours at room temperature. The samples were then transferred to 70% ethanol solution at room temperature. Blood samples were also evaluated by UPLC/MS/MS for quantification of estrogens and progestogens, as previously described, at the Magee-Womens Research Institute (14).

Serum progesterone level was evaluated by the hospital clinical laboratory. All subsequent analyses of IFN-ε and related parameters, including gene expression analysis and IHC quantification, were conducted by researchers in the Hudson Institute of Medical Research in Melbourne, Australia, while blinded to cycle stage of all participants to ensure completely unbiased analysis was conducted. Once all analysis techniques and quantification were complete, researchers at the Magee-Womens Research Institute informed the researchers at the Hudson Institute of the cycle stage of each participant and subsequent graphing and statistical analysis were performed.

#### Cohort 2.

For primary uterine epithelial cell cultures for ex vivo studies, human endometrial specimens were obtained from 4 ovulating women in the proliferative stage of the menstrual cycle who were undergoing hysterectomy or endometrial biopsy for nonendometrial benign pathologies at Monash Health following written informed consent and with approval from the Monash Health Human Research Ethics Committee. Endometrial cells were isolated from donors on the day of sampling by mincing, digestion with collagenase and DNase I, and filtration, as previously described (8). Uterine epithelial cells were cultured for 3 days in phenol-free DMEM-F12 (Gibco, Thermo Fisher Scientific) supplemented with 10% vol/vol charcoal-stripped FCS and 1% penicillin/streptomycin prior to use in experiments.

### Cell culture and reagents

We performed a series of in vitro experiments to determine the response to IFN-ε of various hFRT-derived cells. VK2 (vaginal; ATCC CRL-2616), Ect1 (ectocervical; ATCC CRL-2614), and End1 (endocervical; ATCC CRL-2615) cell lines were maintained in keratinocyte-SFM (Thermo Fisher Scientific), supplemented with 0.2 ng/mL human recombinant EGF, 20 μg/mL recombinant bovine pituitary extract, and 1% Antibiotic-Antimycotic (Gibco, Thermo Fisher Scientific). ECC-1 endometrial epithelial cells (ATCC) were cultured in DMEM:F12 Glutamax (Gibco, Thermo Fisher Scientific) supplemented with 1% penicillin/streptomycin and 10% vol/vol FBS, and validity was routinely tested using short tandem repeat DNA profiling of human cell lines per ATCC guidelines. For stimulation, cells were plated at 1.5 × 10^5^ cells/well in a 12-well plate and stimulated for 3 hours with 100 IU/mL recombinant IFN-ε (made as described in ref. 15) or IFN-β (REBIF, Merck).

### Reporter gene assays

The human *IFNE* promoter (ATG; 1,200 bp) was cloned into the NheI/XhoI restriction site of the luciferase-reporter plasmid, promoter-less pGL3 basic vector (Promega). Cells from an endometrial cancer cell line, ECC-1 (ATCC CRL-2923), were plated in a 96-well plate (2 × 10^4^/well) 24 hours prior to transfection with 60 ng of *IFNE* promoter construct using Lipofectamine 3000 reagent (Thermo Fisher Scientific). Thymidine kinase *Renilla* was used to normalize for transfection efficiency and an appropriate pEF-BOS empty vector plasmid was used to maintain a constant amount of DNA. Transfected cells were lysed using Reporter Lysis Buffer (Promega) and assayed for luciferase and *Renilla* activity using a luciferase assay reagent (Promega) and *Renilla* substrate. Luminescence readings were detected using FLUOstar Optima (BMG Technologies) corrected for *Renilla* and expressed as fold induction over empty vector control values.

### Gene expression analysis

RNA was extracted using TRIzol reagent (Invitrogen, Thermo Fisher Scientific) and the RNeasy Kit (QIAGEN) from biopsy samples obtained from the cross-sectional cohort described above. cDNA was synthesized after DNase treatment (Promega) using Moloney murine leukemia virus reverse transcriptase and random hexamers (Promega).

#### Biomark Fluidigm qPCR.

Gene expression was analyzed using the Biomark Fluidigm system. Ct values from the Biomark qPCR were calculated using the Biomark Fluidigm Real-Time PCR Analysis Software. Comparative analysis was performed using RStudio with the HTqPCR package (16), including methods for principal component analysis, visualization, and Spearman’s correlation. Results were normalized to the housekeeping genes *HMBS* and *RPLPO*, which were confirmed to be stably expressed across sample types, and the average of these genes was used to normalize gene expression data. Unreliable probes were removed as defined by postnormalized Ct values > 10 or undetected in greater than 30% of samples. TaqMan probe (Thermo Fisher Scientific)identifiers for genes analyzed are listed in Supplemental Table 1.

#### SYBR green qPCR.

For in vitro studies using cell lines, RNA was extracted as described above and qPCR was performed using SYBR green (Thermo Fisher Scientific) on an Applied Biosystems 7900HT Fast Real-Time PCR machine (Thermo Fisher Scientific). Primer sequences are listed in Supplemental Table 2. Expression was calculated using the 2^−ΔΔCT^ method using *18S* as the endogenous control and relative to control conditions.

### ELISA

Expression levels of IFN-ε in CVL of the 32 participants were determined using a validated prototype sandwich ELISA codeveloped in collaboration with PBL Assay Science. Wells of a 96-well microtiter plate were coated with 1 μg/mL anti–human IFN-ε mAb (HE28 made in-house) in 50 mM carbonate/bicarbonate coating buffer pH 9.5 at 4°C, 16 hours. The plate was blocked with PBS pH 7.4 and 1% BSA at room temperature for 2 hours. Thereafter, the plate was washed 3 tines with PBS pH 7.4 and 0.1% Tween 20. Plates were then incubated with IFN-ε standard (serial dilutions ranging from 3.9 to 250 pg/mL) or a sample (diluted 1:2) in blocking buffer with 0.1% Tween and incubated at 25°C, 450 rpm for 1 hour. The plate was washed as above, and biotinylated anti–human IFN-ε capture mAb (anti–IFN-ε, HE2) was added and incubated at 25°C, 450 rpm for 1 hour. Thereafter, the plate was washed 3 times before adding streptavidin–horseradish peroxidase conjugate and incubated for a further 1 hour, at 25°C, 450 rpm. The plate was washed 4 times, and 3,3′,5,5′-tetramethylbenzidine substrate was added. The plate was incubated at ambient room temperature, protected from light, for 30 minutes. The reaction was terminated using a sulfuric acid solution, and OD 450 nm readings were obtained using a BMG Labtech FLUOstar omega plate reader. Expression of IL-15 and IL-6 in CVL samples from 32 participants was determined using Luminex multiplex assays. CVL was diluted 1:1 in assay buffer and analyzed using a human cytokine assay panel (Merck) per manufacturer’s instructions. Supplied standards and quality controls were included. All assays were performed in duplicate with overnight incubation at 4°C and read using a Bioplex 200 analyzer (Bio-Rad). Mean concentrations were interpolated from a 5-parameter fit standard curve.

### IHC analysis and quantification

Multicore tissue arrays were created at the Monash University Histology Platform from hFRT biopsy samples from the Pittsburgh cohort. Each array contained matched vaginal, ectocervical, and endometrial tissue from up to 9 participants included in this study in order of recruitment into the study and prior to knowledge of the stage of menstrual cycle of the participants. Thin sections (~4 μM) of each tissue array block were cut and adhered onto Superfrost Plus glass slides (4951PLUS4, Thermo Fisher Scientific). Sections were dehydrated using a series of 100% xylene, 100% ethanol, 70% ethanol, and MilliQ water (MilliporeSigma) solutions. Heat-induced antigen retrieval was performed using a 10 mM Trizma base (T6066, MilliporeSigma) and 1 mM EDTA buffer at pH 9.0. Sections were blocked using CAS-block (00-8120, Invitrogen, Thermo Fisher Scientific), for 1 hour at room temperature, then incubated overnight at 4°C with the following primary Abs: rabbit anti–human IFN-ε (NBP1-92018, Novus Biologicals), used at 0.5 μg/mL; mouse anti–human estrogen receptor α (IR657, Dako), provided at ready-to-use concentration; and mouse anti–human PR (M3569, Dako), used at 1.56 μg/mL; all diluted in CAS-block. Corresponding isotype controls were rabbit IgG (I-1000, Vector Laboratories), used at 0.5 μg/mL; and mouse IgG1 (X0931, Dako), used at 1.56 μg/mL; both diluted in CAS-block. Sections were washed with 0.05% Tween-PBS for 15 minutes and incubated with 60 μg/mL biotinylated secondary Abs (BA-1000, anti-rabbit; and BA9200, anti-mouse; Vector Laboratories) for 1 hour at room temperature. Slides were washed in 0.05% Tween-PBS for 10 minutes, incubated for 45 minutes with the VECTASTAIN Elite ABC-HRP Kit, an avidin-biotinylated peroxidase H complex (PK-6100, Vector Laboratories), and washed again for 10 minutes in 0.05% Tween-PBS, and DAB substrate (GV825, Dako) was applied for 30 seconds to initiate precipitate formation/color development via peroxidase activity. Enzyme activity was stopped using distilled water. Coverslips were applied to slides with DPX mounting media (Merck) and allowed to dry overnight.

### Slide scanning and image analysis

High-resolution digital scans were acquired using the Aperio Scanscope AT Turbo (Leica Biosystems) at the Monash Histology Platform. Quantification was performed using Aperio ImageScope (version 12.3.0.5056, Leica Biosystems) with the Aperio Cytoplasm Algorithm (version 2, Leica Biosystems). The epithelium, glands, and stroma were delineated and assessed independently of each other for all staining. For quantification of IFN-ε expression by IHC, we used the positive pixel count (version 9) algorithm to measure the intensity of the marker (brown signal) in epithelial and stromal areas of tissue for each participant. The positive-count algorithm, which quantifies the amount of a specific stain present in a scanned slide image, was used to determine relative intensity values.

Because hormone receptor staining showed distinct cytoplasmic and nuclear staining patterns, an H-score for nuclear and cytoplasmic staining was obtained using the Aperio Nuclear and Cytoplasmic algorithms; each of these calculates an H-score based on the following classification of staining as follows: 0, none; 1+, weak; 2+ moderate; 3+, strong and then generation of the H-score using the following formula: 1 × (%1+) + 2 × (%2+) + 3 × (%3+); therefore, a maximum H-score of 300 would indicate that 100% of cells stained as 3+. All quantification was performed by researchers while blinded to the cycle stage of the participants.

### Statistics

For Fluidigm analysis, statistically significant gene changes were determined between conditions using linear modeling, using the *limma* statistical package (17). An empirical Bayes moderated *t* test was used, with a 1.5-fold change cutoff and Benjamini-Hochberg correction for false discovery. Gene expression was considered significant at *P* < 0.05. For all other data, statistical analyses were performed using GraphPad Prism (version 9.3.0, GraphPad Software). Mann-Whitney *U* tests and Kruskal-Wallis testing with Dunn’s multiple-comparison analysis were used as indicated. Spearman’s rank correlation coefficient analysis was used for all correlation analysis.

### Study approval

The University of Pittsburgh IRB (cohort 1) and the Monash Health Human Research Ethics Committee (Clayton, Victoria, Australia) (cohorts 1 and 2) approved this study. Written informed consent was collected from each participant prior to study participation.

## Author contributions

NMB led the experimental work and contributed to planning and writing the manuscript; SLA led the planning, execution, and interpretation of clinical studies performed at University of Pittsburgh Magee-Womens Research Institute and contributed to writing the manuscript; SUH processed samples and performed and interpreted IHC experiments; HEC, LJG, and RC contributed to the design and analysis of gene expression arrays; SSL contributed to the measurements of IFN-ε by ELISA; IP performed in vitro type I IFN experiments; ST conducted experimental work and developed protocols under the supervision of SM; NEM contributed to experimental and conceptual aspects of the project; SM contributed to planning and interpretation of hormone data and contributed to writing the manuscript; PJH led the intellectual development of the overall project and contributed to interpretation of data and writing the manuscript.

## Supplementary Material

Supplemental data

## Figures and Tables

**Table 1:**
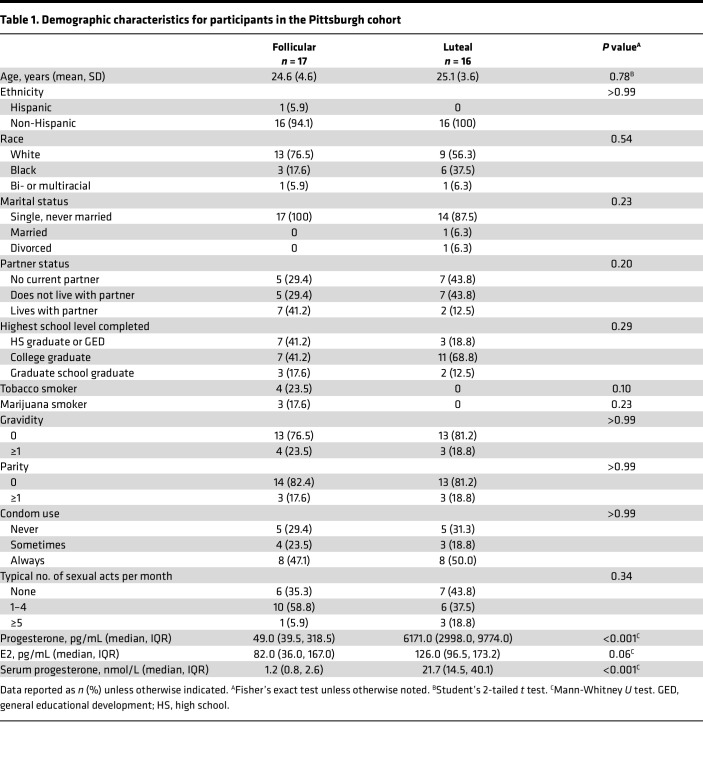
Demographic characteristics for participants in the Pittsburgh cohort

## Version 1 07/21/2022

In-Press Preview

## Version 2 09/22/2022

Electronic publication
