# Supplementary material for: Spatiotemporal regulation of human IFN-ε and innate immunity in the female reproductive tract
Source: JCI Insight. 2022 Sep 22;7(18):e135407. doi: 10.1172/jci.insight.135407 (PMC9675573; doi:10.1172/jci.insight.135407)
Supplement: Supplemental data [file jciinsight-7-135407-s162.pdf]

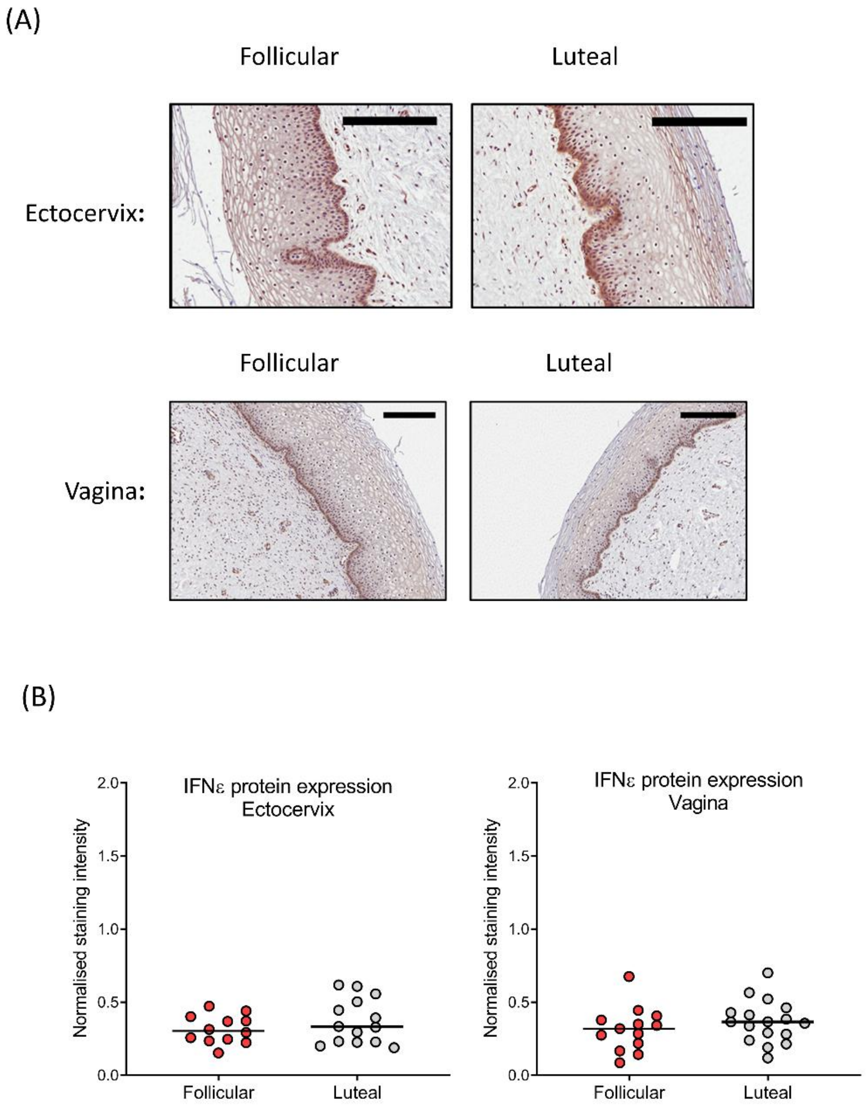

Figure S1. (A) Representative IHC images of IFNε (brown) in vaginal and ectocervical sections in the follicular and luteal phase of menstrual cycle. Bar, 200 μM. (B) Quantification of ectocervical and vaginal epithelial IFNε staining intensity in women in follicular or luteal stage of menstrual cycle.

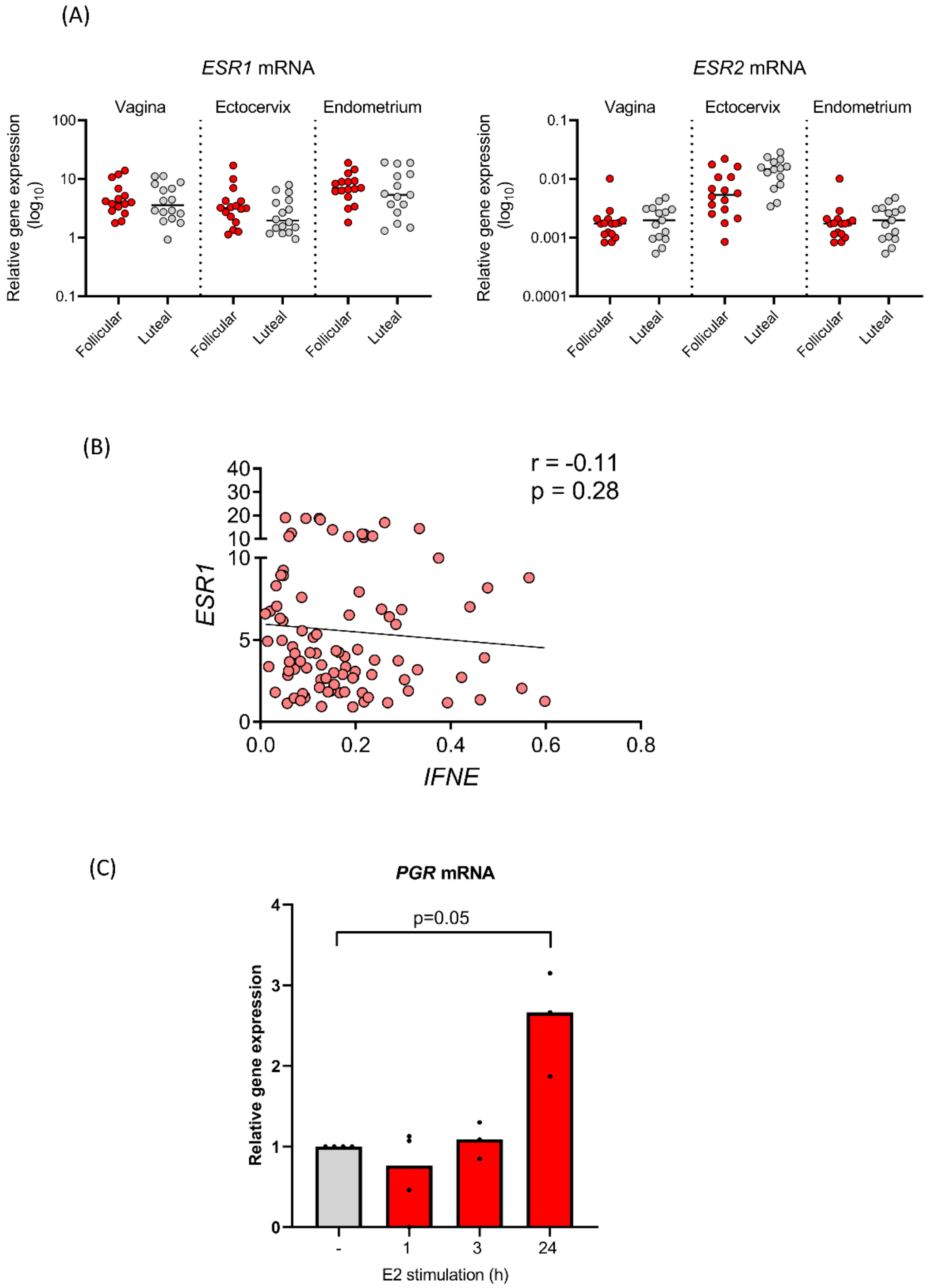

Figure S2. (A) Expression of *ESR1* and *ESR2* in vaginal, ectocervical and endometrial biopsies as determined using qPCR. Expression stratified by stage of menstrual cycle and median expression is shown. Data analysed using Kruskal-Wallis testing with Dunn's multiple comparison analysis. (B) Spearman correlation analysis of *ESR1* and *IFNE* expression in the FRT. (C) PGR mRNA expression in primary uterine epithelial cells from n=3 donors stimulated with 10nM estrogen for up to 24h. Data analysed using Kruskal-Wallis testing with Dunn's multiple comparison analysis.

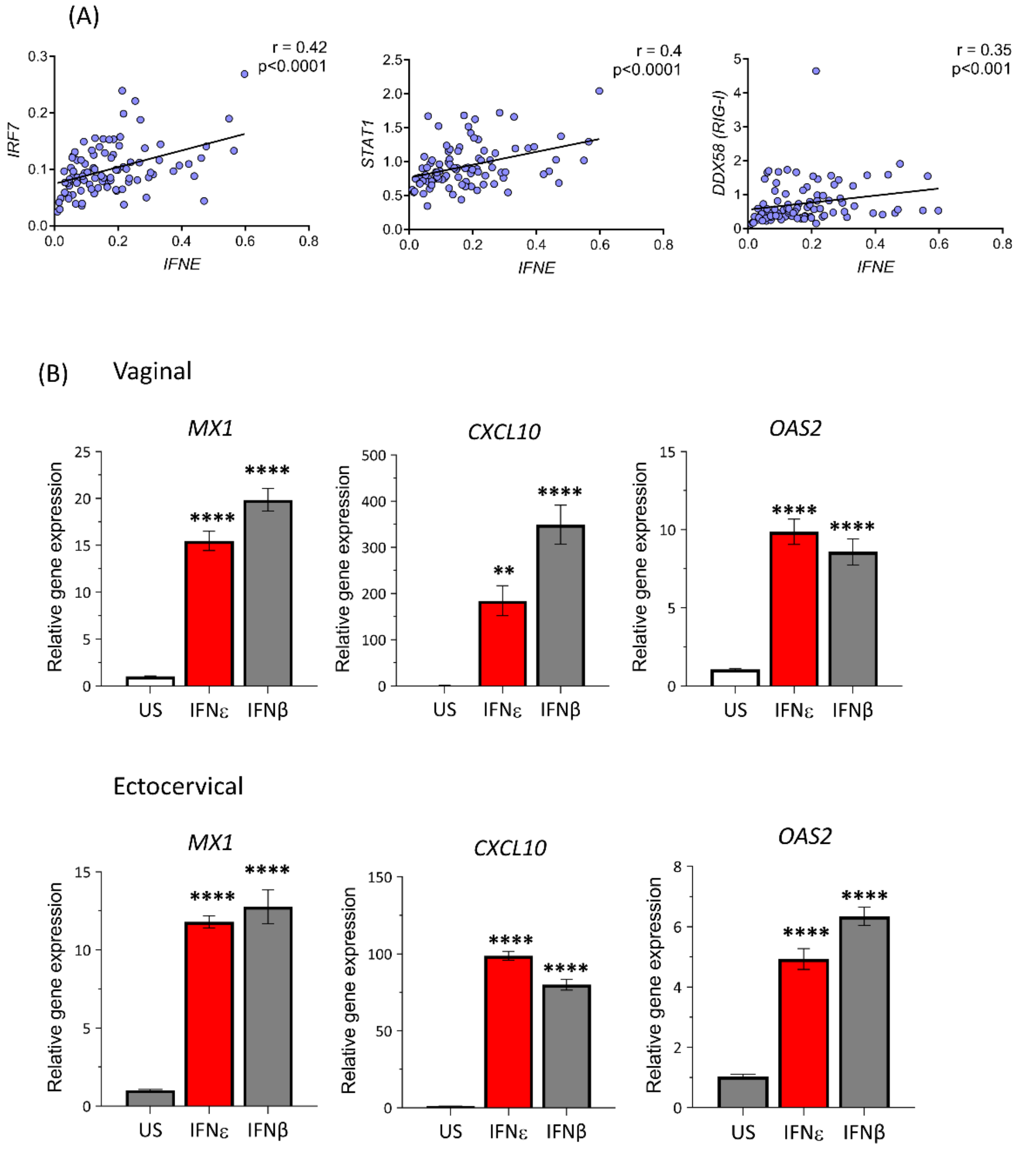

Figure S3. (A) Spearman correlation analysis of the expression of *IFNE* with the IRGs, *IRF7*, *STAT1* and *DDX58* across FRT samples. (**B**) Vaginal (VK2) and ectocervical (Ect1) cells were stimulated for 3hrs with 100 IU/ml of recombinant IFNε or IFNβ and expression of the IRGs *MX1*, *CXCL10* and *OAS2* were quantified by qPCR. Expression relative to unstimulated control (US). Data from n=3 independent biological replicates, shown as mean ±SEM and analysed using one-way ANOVA with Dunnett's multiple comparisons testing **p<0.01, ****p<0.0001.

Supplemental Table 1: Taqman probe IDs

**Gene name**	**Taqman ID**
IFNA1	Hs00855471_g1
IFNA2	Hs00265051_s1
IFNA4	Hs01681284_sH
IFNB	Hs01077958_s1
IFNL1	Hs00601677_g1
IFNL2	Hs00820125_g1
IFNL3	Hs04193047_gH
IFNE	Hs00703565_s1
IFNY	Hs00989291_m1
ESR1	Hs00174860_m1
PGR	Hs01556702_m1
ESR2	Hs00230957_m1
18S	Hs99999901_s1
RPLPO	Hs00420895_gH
HMBS	Hs00609296_g1
MX1	Hs00895608_m1
OAS2	Hs00942643_m1
CXCL10	Hs01124251_g1
DDX58	Hs00204833_m1
IRF7	Hs01014809_g1

Supplemental Table 2: Primers for SyBr green qPCR

	**F**	**R**
18S	GTAACCCGTTGAACCCCATT	CCATCCAATCGGTAGTAGCG
MX1	GGTGGTGGTCCCCAGTAATG	ACCACGTCCACAACCTTGTCT
CXCL10	TTCCTGCAAGCCATTTTGT	TTCTTGATGGCCTTCGATTC
OAS2	GAAGCCCTACGAAGAATGTCAGA	TCGGAGTTGCCTCTTAAGACTGT
